# COSMC knockdown mediated aberrant O-glycosylation promotes oncogenic properties in pancreatic cancer

**DOI:** 10.1186/s12943-015-0386-1

**Published:** 2015-05-29

**Authors:** Bianca T. Hofmann, Laura Schlüter, Philip Lange, Baris Mercanoglu, Florian Ewald, Aljonna Fölster, Aeint-Steffen Picksak, Sönke Harder, Alexander T. El Gammal, Katharina Grupp, Cenap Güngör, Astrid Drenckhan, Hartmut Schlüter, Christoph Wagener, Jakob R. Izbicki, Manfred Jücker, Maximilian Bockhorn, Gerrit Wolters-Eisfeld

**Affiliations:** Department of General, Visceral and Thoracic Surgery, University Medical Center Hamburg-Eppendorf, Martinistrasse 52, 20246 Hamburg, Germany; Department of Hepatobiliary and Transplant Surgery, University Medical Center Hamburg-Eppendorf, Martinistrasse 52, 20246 Hamburg, Germany; Department of Clinical Chemistry, University Medical Center Hamburg-Eppendorf, Martinistrasse 52, 20246 Hamburg, Germany; Department of Anatomy and Experimental Morphology, University Medical Center Hamburg-Eppendorf, Martinistrasse 52, 20246 Hamburg, Germany; Institute for Biochemistry and Signal Transduction, University Medical Center Hamburg-Eppendorf, Martinistrasse 52, 20246 Hamburg, Germany

**Keywords:** Pancreatic cancer, PDAC, O-GalNAc, O-glycosylation, Tn antigen, COSMC, C1GALTC1, Nucleolin

## Abstract

**Background:**

Human pancreatic ductal adenocarcinoma (PDAC) is one of the most aggressive and lethal malignancies in the world and despite great efforts in research types of treatment remain limited. A frequently detected alteration in PDACs is a truncated O-linked N-acetylgalactosamine (GalNAc) glycosylation with expression of the Tn antigen. Changes in O-glycosylation affect posttranslationally modified O-GalNAc proteins resulting in profound cellular alterations. Tn antigen is a tumor associated glycan detected in 75-90 % of PDACs and up to 67 % in its precursor lesions. Since the role of Tn antigen expression in PDAC is insufficiently understood we analyzed the impact of COSMC mediated Tn antigen expression in two human PDAC cell lines on cellular oncogenic properties.

**Methods:**

Forced expression of Tn antigen on O-glycosylated proteins in pancreatic cancer cells was induced by lentiviral-mediated knockdown of the COSMC chaperone, which prevented O-glycan elongation beyond the initial GalNAcα1- residue on O-linked glycoproteins. Altered O-GalNAc glycosylation was analyzed in human pancreatic cancer cell lines Panc-1 and L3.6pl using Western and Far-Western blot as well as immunocytochemical techniques. To assess the biological implications of COSMC function on oncogenic properties, cell viability assays, scratch assays combined with live cell imaging, migration and apoptosis assays were performed. Lectin based glycoprotein enrichment with subsequent mass spectrometric analysis identified new cancer O-GalNAc modified proteins. Expression of Tn antigen bearing Nucleolin in patient derived PDAC tumor specimens was evaluated and correlated with clinicopathological data.

**Results:**

Tn antigen expression was induced on various O-GalNAc glycoproteins in COSMC deficient cell lines compared to the control. Proliferation was reduced (*p* < 0.001) in COSMC knockdown cells, whereas migration was increased (*p* < 0.001) and apoptosis was decreased (*p* = 0.03), highlighting the importance of Tn antigen expression on metastatic and anti-apoptotic behavior of PDAC derived cells. Nucleolin was identified as O-GalNAc modified protein in COSMC deficient PDAC cell lines. Interestingly, immunohistochemical staining and co-localization studies of patient derived PDACs revealed poor survival for patients with strong co-localization of Tn antigen and Nucleolin (*p* = 0.037).

**Conclusion:**

This study substantiates the influence of altered O-glycan (Tn/STn) expression on oncogenic properties in pancreatic cancer and identifies O-GalNAc modified Nucleolin as novel prognostic marker.

**Electronic supplementary material:**

The online version of this article (doi:10.1186/s12943-015-0386-1) contains supplementary material, which is available to authorized users.

## Background

Changes in O-GalNAc glycosylation (N-acetylgalactosaminyl glycosylation; hereafter referred simply as O-glycosylation) are typical characteristics of malignant transformation in epithelial cells [1]. Given the complexity of protein glycosylation and its superordinated impact on a diverse range of biological processes it is not surprising that seemingly minor alterations in carbohydrate structure can significantly impact cell biology. Current research on differential O-glycosylation starts to elucidate its influence on carcinogenesis and malignant transformation. For instance, O-glycosylation of DR4/DR5 death receptor regulates apoptosis resistance or sensitivity [2] and aberrant O-glycosylation of β-integrins alters the cellular phenotype and influences proliferation and haptotaxis [3]. Recently, Radhakrishnan *et al.* demonstrated the impact of truncated O-glycans on cell-cell adhesion and migration in pancreatic cancer [4].

**Fig. 1:**
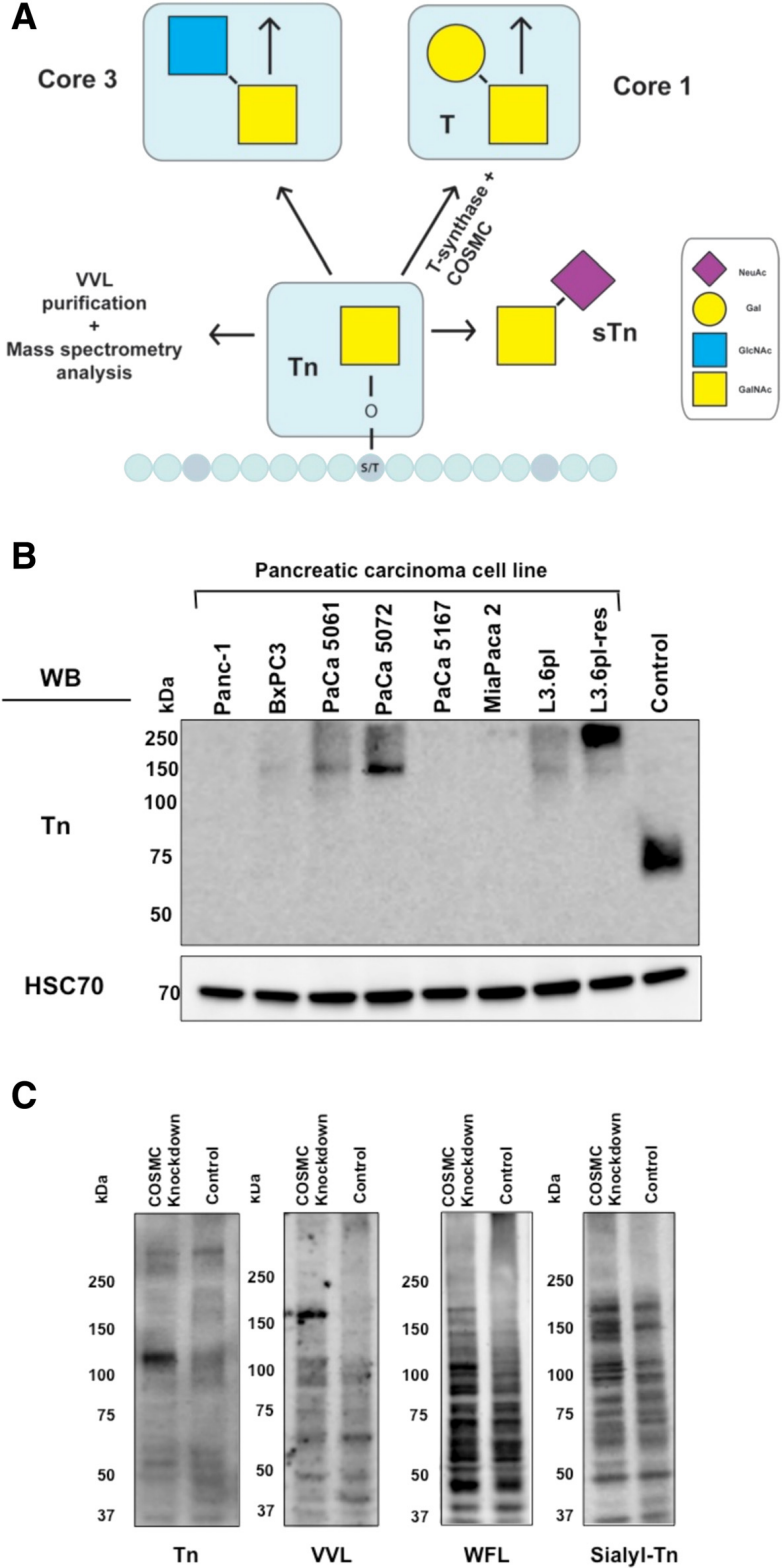
Expression of aberrant O-glycans in pancreatic cancer. **a** Biosynthesis of Tn antigen, sTn antigen and Core1 and 3 structures. Tn antigen is composed of an O-glycosidic linked *N*-acetylgalactosamine (GalNAc) to the –OH group of serine/threonine (S/T). Tn antigen is either processed by core 1 T-synthase (C1GalT1) and its chaperone (COSMC), which transfers a galactose (Gal) to GalNAc-serine/threonine to form the T antigen also referred as core 1 structure or processed by transfer of a *N*-acetylglucosamine (GlcNAc) to form the core 3 structure. Tn antigen can also be modified by addition of a sialic acid (NeuAc). **b** Differential expression of Tn antigen in pancreatic carcinoma cell lines. Eight different PDAC cell lines were available for analysis. Western and Far-Western blot analysis of total cell lysates was performed using the Tn antigen specific antibody MA1-80055. Detection of HSC70 served as loading control. Jurkat cells were used as positive control for Tn antigen expression. **c** Expression of Tn antigen and aberrant O-glycans in COSMC knockdown cells. Western blot analysis showed a strong expression of aberrant O-glycans as well as Tn antigen in Panc-1 COSMC knockdown cells compared to control cells. Sialyl-Tn and Tn antibodies were used as well as lectins such as VVL (*Vicia villosa* lectin) and WFL (*Wisteria floribunda* lectin)

The Tn antigen (GalNAcα1-O-Ser/Thr) is a frequently occurring aberrant O-glycan expressed at high levels in many cancers [5] including pancreatic ductal adenocarcinoma (PDAC) [6–8], PDAC precursor lesions [7] and is detectable in PDAC sera [9, 10]. Expression of Tn antigen and its sialylated form sialyl-Tn (STn) antigen is associated with poor survival [11, 12] and promotes oncogenic features [4]. Tn antigen expression is initiated by polypeptide N-acetylgalactosaminyltransferases (GalNAc-Ts), which connect GalNAc residues with the target protein as a posttranslational modification. In humans, 20 different GalNAc-Ts are identified so far, leading to a complex interplay of various enzymes reflected in the cellular O-glycobiome. Tn antigen is further processed by core 1 synthase (C1GALT1 or T-synthase), which transfers Galactose (Gal) to GalNAc-Ser/Thr to form the T antigen, also referred as core 1 structure. COSMC (C1GALT1C1) is the unique chaperone of T-synthase and is essential for its functional formation in order to elongate glycans beyond the initial Tn structure (Fig. 1a) [13]. Dysfunctional COSMC is also able to convert a wild type protein into a tumor-specific antigen [14] affecting tumor cell biology.

Several factors are known to contribute to the formation of Tn/STn antigen on glycoproteins. Major factors are altered levels in *COSMC* and/or *T-synthase* gene expression as well as differential expression and localization of GalNAc-transferases. In detail, acquired mutations in *COSMC* [13, 15], epigenetic silencing of *COSMC* and/or *T-synthase*, including hypermethylation of the *COSMC* promotor [4, 16] as well as altered signaling pathways and altered expression or localization of GalNAc-transferases [17–22] may contribute to Tn and/or STn expression.

Tn/STn antigens are cancer-associated glycans recognized by the human macrophage galactose binding glycoreceptor MGL (CLEC10A/CD301) [23–25] that is expressed on immature and tolerogenic dendritic cells and macrophages. Since Tn/STn glycans and glycopeptides are not or poorly immunogenic, vaccination based cancer therapy remains challenging. Nonetheless, the attempt of generating Tn specific antibodies with *in vivo* anti-tumor activity was described [26, 27]. Preclinical animal studies showed promising immunogenicity, but none of the immuno conjugates succeeded in clinical trials, despite safe administration and proper immune responses [28]. Recent and ongoing vaccine trials are encouraging for future trials and the design of proper immunogens and immuno-conjugates remain the main challenge. Interestingly, ABO blood group IgM agglutinins/antibodies were observed to interact with PDAC O-GalNAc modified glycoproteins possibly affecting cancer onset [29].

Nevertheless, pancreatic carcinoma is one of the worlds’ most aggressive malignancies [30] and consequences of COSMC mediated Tn antigen expression in pancreatic carcinoma are not fully understood. Investigation of Tn modified glycoproteins and its impact on oncogenic properties is crucial to understand tumor biology and potential therapeutic options.

## Results

### Differential expression of Tn antigen in human pancreatic carcinoma cell lines

Several PDAC derived cell lines were available for Tn antigen screening using Western and Far-Western blot analysis. Besides commercially available PDAC cell lines such as Panc-1, BxPC3, MiaPaca2 and L3.6pl, patient derived cell lines PaCa 5061 [31], PaCa 5072 and PaCa 5167, and a Gemcitabine resistant sub-clone of the parental L3.6pl cell line, L3.6pl-res cells were used [32]. Analysis of Tn antigen expression in cell lysates was performed using distinct antibodies or glyco epitope recognizing lectins. Jurkat cells express mutated COSMC [13] and were used as Tn antigen positive control. Interestingly, PDAC cell lines displayed differential expression of the Tn antigen. Using the Tn antigen specific antibody MA1-90544, L3.6pl-Res and PaCa 5072 cells showed a strong positivity, whereas L3.6pl, PaCa 5061 and BxPC3 cells were weakly positive. Minimal signals were detectable in Panc-1, PaCa 5167 and MiaPaca2 cells (Fig. 1b). Similar results were obtained using VVL, HPA lectins and sialyl Tn antibody reflecting consistency (Additional file 1: Figure S1-S3).

**Fig. 2:**
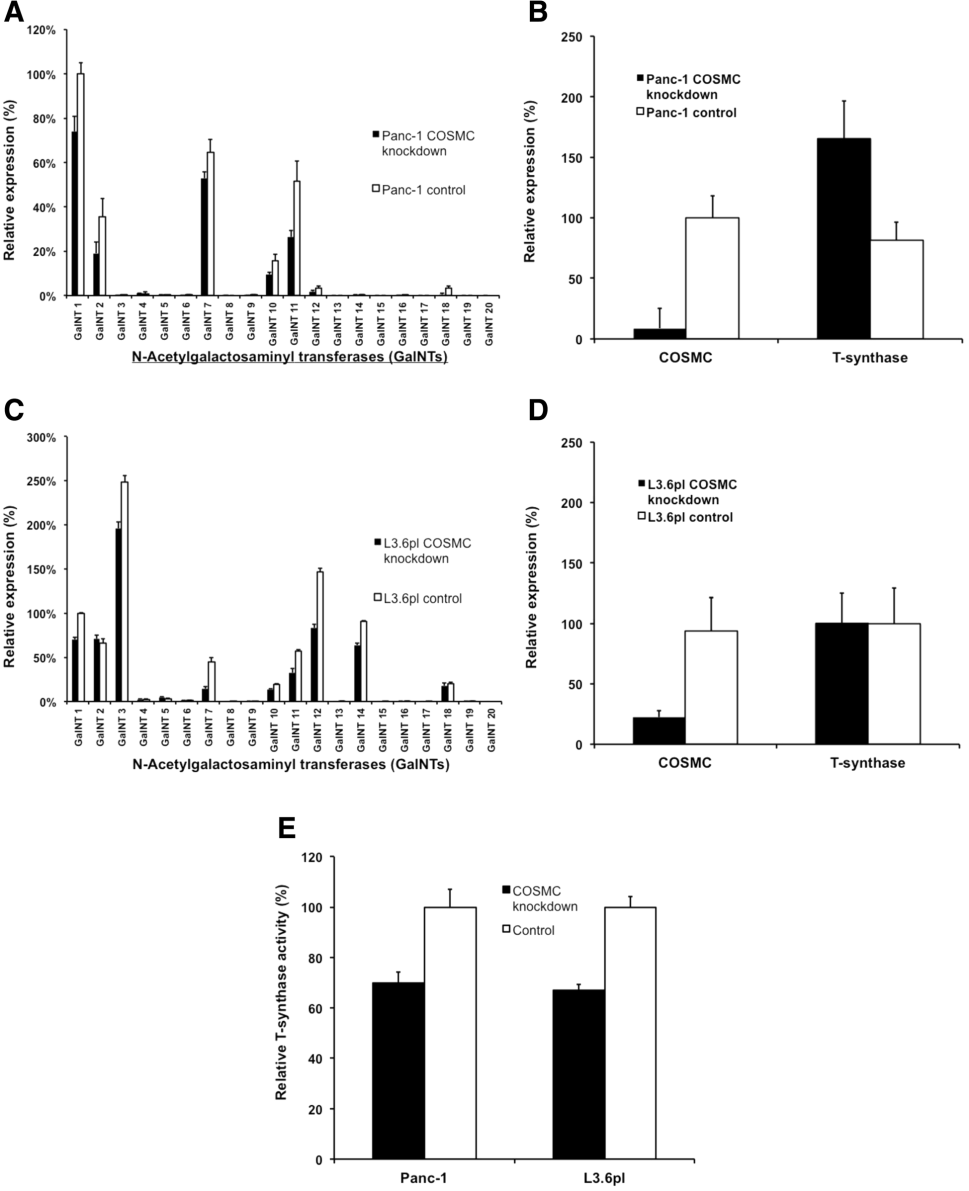
Aberrant mRNA expression levels of GalNAc-transferases, *T-Synthase* and *COSMC* as well as T-synthase activity in COSMC-depleted pancreatic cancer cells. **a** Relative mRNA expression of all human GalNAc-transferase isoforms in COSMC knockdown as well as control Panc-1 cells, quantified using real-time PCR. **b** Relative quantification of T-synthase mRNA expression in COSMC knockdown and control Panc-1 cells. Note: mRNA expression level of T-synthase is almost doubled in COSMC knockdown cells. **c** Relative mRNA expression of GalNAc-transferase isoforms in L3.6pl. **d** Relative quantification of T-synthase and COSMC mRNA expression in L3.6pl COSMC knockdown and control cells. **e** T-synthase activity was measured in Panc-1 and L3.6pl COSMC knockdown and control cells using GalNAc-α-4MU fluorescent assay

In order to induce the expression of Tn antigen in the minimal Tn antigen positive Panc-1 cell line and the moderate Tn antigen positive L3.6pl cell line, we performed a lentiviral-mediated knockdown of COSMC chaperone followed by single colony selection with puromycin. Knockdown of COSMC was highly efficient in Panc-1 cells, compared to cells transduced with a control vector (Additional file 1: Figure S4). Additionally, down regulation of COSMC was confirmed on mRNA level by real-time PCR, achieving 97 % efficiency in Panc-1 and 73 % in L3.6pl cells (Fig. 2b and d). Next, expression of atypical O-glycans was evaluated using different glyco epitope recognizing antibodies and lectins. Using sialyl-Tn and Tn antigen antibodies as well as VVL and WFL, revealed an enhancement of distinct staining patterns in COSMC knockdown cells compared to control cells (Fig. 1c). L3.6pl cells revealed an enhanced Tn antigen expression only after treatment with Neuraminidase (Additional file 1: Figure S5). Immunocytochemistry revealed an enhanced staining of VVL and WFL lectin in COSMC knockdown cells compared to controls (Additional file 1: Figure S6).

### Decreased expression of GalNAc-transferases in COSMC knockdown cells

COSMC knockdown causes a cell type specific Tn antigen expression pattern mediated by the subset of expressed GalNAc-transferases. Since GalNAc-transferase 2 and 5 are already identified as O-GalNAc modified glycoproteins [33], a COSMC knockdown could influence GalNAc-transferase activity and consecutive GalNAc-T expression levels as a cellular response. This could result in an altered Tn antigen expression pattern caused by impaired GalNAc-T activity compared to the initially induced COSMC knockdown Tn antigen expression pattern. Since GalNAc-transferase activity assays are not available, we analyzed GalNAc-Ts expression levels in COSMC knockdown cells compared to controls. We used quantitative real-time PCR to evaluate expression levels of all known human GalNAc-Ts (1–20) in Panc-1 as well as L3.6pl COSMC knockdown cells and corresponding control cells. In seven out of 20 investigated GalNAc-T isoform mRNAs were readily detectable using real-time PCR and showed differential mRNA expression levels in COSMC knockdown cells. The GalNAc-T isoforms 1, 2, 7, 10, 11, 12, and 18 were expressed in Panc-1 cells (Fig. 2a). Interestingly, COSMC-depleted Panc-1 cells showed a substantial decrease in mRNA expression for all detected GalNAc-T isoforms, compared to the respective control cells. In fact, relative mRNA expression was decreased up to 50 % (*p* <0.001) (Fig. 2a). Also, analysis of mRNA expression levels of the T synthase, a key enzyme in the cellular O-glycan pathway, revealed a substantial increase in COSMC knockdown cells, compared to control cells (Fig. 2b). L3.6pl cells showed the expression of GalNAc-T isoforms 1, 2, 3, 7, 10, 11, 12, 14 and 18 (Fig. 2c). Except GalNAc-T 2, all other isoforms showed decreased mRNA expression in COSMC knockdown cells, comparable to the results of Panc-1 COSMC knockdown and control (Fig. 2c). In contrast, L3.6pl COSMC knockdown was approximately 80 %, and T-synthase levels did not display any differences (Fig. 2d). Next, T-synthase activity was measured in Panc-1 and L3.6pl cells. Panc-1 COSMC knockdown cells displayed 69.9 % T-synthase activity and T-synthase activity in L3.6pl COSMC knockdown cells was 67 %, compared to control cells (Fig. 2e).

### MS-based identified Nucleolin, GRP-78, α-Enolase and Annexin A2 display O-GalNAc glycosylation

Although several Tn antigen carrier molecules have been identified in various cancers, the molecular identity of these different O-glycosylated proteins still remains enigmatic. In order to identify new pancreatic cancer specific O-glycosylated proteins that express the Tn antigen, we applied lectin-based immunoprecipitation using VVL in COSMC knockdown as well as corresponding control cells (Additional file 1: Figure S7) accompanied by peptide identification *via* mass spectrometry. VVL is able to bind glycopeptides through single GalNAc molecules and its binding affinity was demonstrated to depend on Tn antigen cluster formation [34].

Among the top hits, we identified Nucleolin, GRP-78, Annexin A2 and α-Enolase (Tab.1). A careful review of the literature revealed that Nucleolin and GRP-78 have previously been described to carry O-glycosylated residues [33, 35]. These four candidates appeared to be promising, because these proteins i) are differentially expressed in pancreatic carcinoma [36–39], ii) are cell membrane localized (at least in carcinoma) [40–43] iii) and are known to promote cancer cell signaling. Moreover, *in silico* analysis of the identified peptide protein sequences using the isoform specific O-glycosylation prediction (ISOGlyP) database [44], revealed that these proteins are likely to be O-GalNAc glycosylated [33]. Referring to the results of GalNAc-transferase isoform expression of Panc-1 cells, isoforms T1 and T12 showed highest prediction possibility of O-GalNAc-glycosylation for Nucleolin. Highest prediction for GRP-78 showed GalNAc-transferase isoforms T2 and T12, for α-Enolase T10 and T12 and for Annexin A2 T1 and T2.

**Table 1:** Mass spectrometry identified O-GalNAc modified proteins

Protein	Gene	Uniprot acc. No.	NetOGlyc 4.0	References
Nucleolin	NCL	P19338	+++	[35]
Alpha-1-acid glycoprotein 1	AGP1	P02763	-	
Alpha-actinin-4	ACTN4	O43707	+++	
Ig alpha-1 chain C	IGHA1	P01876	++	
Elongation factor 2	EEF2	P13639	++	
GRP-78	HSPA5	P11021	+	[33]
HSP73	HSPA8	P11142	+	
PABP1	PABPC1	P11940	++	
PABP4	PABPC4	Q13310	+	
Alpha-enolase	ENO1	Q6GMP2	+	
Elongation factor 1-gamma	EEF1G	P26641	++	
EF-1-alpha-like 3	EEF1A1P5	Q5VTE0	+	
Tubulin beta	TUBB2A	Q13885	-	
Galectin-12	LGALS12	Q96DT0	++	
Ras association domain-containing protein 2	RASSF2	P50749	+++	
GAPDH	GAPDH	P04406	+	
Nucleophosmin	NPM1	Q8WTW5	+++	
Annexin A2	ANXA2	P07355	+	
37 kDa laminin receptor precursor	RPSA	Q86VC0	+++	
Leucyl-cystinyl aminopeptidase	LNPEP	Q9UIQ6	+	
eIF-5B	EIF5B	O60841	+++	
Armadillo repeat-containing protein 4	ARMC4	Q5T2S8	+++	
LanC-like protein 2	LANCL2	Q9NS86	++	

Proteins identified from Panc-1 COSMC knockdown cells by immunoprecipitation using VVL agarose accompanied by mass spectrometry. The O-GalNAc prediction tool (NetOGlyc 4.0) [52] was used to assess possibility of O-glycosylation. Score indicates percentage of possibly O-GalNAc modified Serin/Threonin residues (+ <10 %, ++ <20 %, +++ > 20 %). References are presented for previously identified O-GalNAc modified glycoproteins

**Fig. 3:**
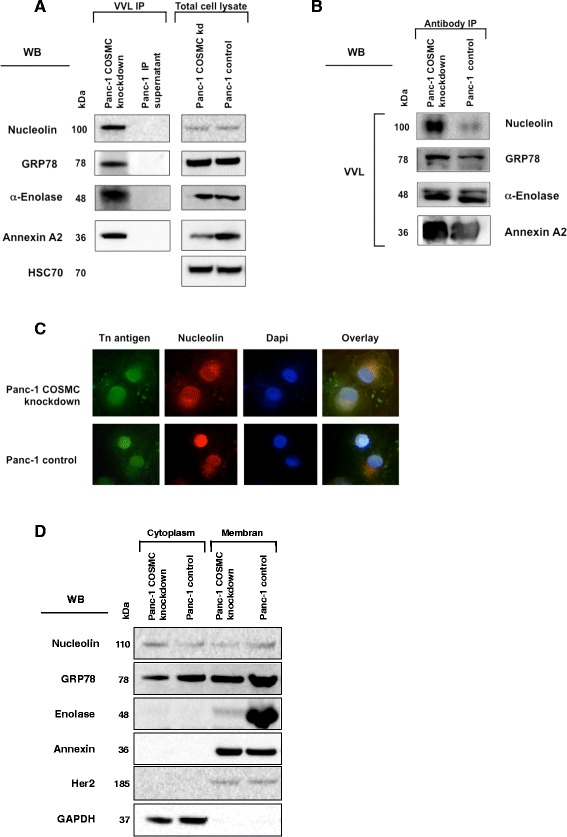
MS identified Nucleolin, GRP-78, α-Enolase and Annexin A2 display O-GalNAc glycosylation. **a** *left*: Tn antigen is expressed on Nucleolin, GRP-78, α-Enolase and Annexin A2. Immunoprecipitation and western blot analysis were performed using VVL lectin for Tn antigen precipitation and Nucleolin, GRP-78, α-Enolase and Annexin A2 antibodies for detection of specific protein signals. **a** *right*: Protein expression levels of the identified proteins in total cell lysates of COSMC knockdown as well as control cells remained unchanged, except for Annexin 2, which showed a decrease in Panc-1 COSMC knockdown cells. **b** Vice versa, immunoprecipitation using specific antibodies and detection with VVL lectin was performed as well. **c** Immunocytochemistry of Panc-1 COSMC knockdown cells and control cells. *Left*: Aberrant O-glycans, including Tn antigen, were detected using VVL-FITC conjugated lectin (*green*). *Middle left*: Nucleolin was detected (*red*) using an anti-Nucleolin antibody (ab13541). *Middle right:* Nuclei were stained with DAPI (*blue*). *Right*: Overlay of Tn antigen and Nucleolin immunocytochemistry. **d** Nucleolin, GRP-78, Enolase and Annexin detection in the cell membrane protein fraction. Her2 antibody was used as marker for membrane fraction and GAPDH antibody was used as marker for cytoplasmic proteins

For proof-of-concept, we confirmed the Tn expression on Nucleolin, GRP-78, α-Enolase and Annexin A2 through VVL-based immunoprecipitation followed by detection of the target proteins with specific antibodies (Fig. 3a). Protein expression levels of the identified proteins in total cell lysates of COSMC knockdown as well as control cells remained unchanged, except for Annexin A2 which showed a decrease in Panc-1 COSMC knockdown cells (Fig. 3a). *Vice versa* immunoprecipitation with the protein specific antibody and detection with VVL lectin showed distinct staining patterns at indicated molecular weight (Fig. 3b). Additionally, we performed immunocytochemistry to investigate the intracellular localization of Tn antigen and Nucleolin using COSMC depleted Panc-1 cells. Interestingly, VVL mediated Tn antigen staining showed cell surface as well as a predominant cytoplasmic staining. The co-staining of Nucleolin in COSMC knockout Panc-1 cells revealed also cell membrane localization and a nuclear/perinuclear staining. Co-staining of Tn antigen and Nucleolin in COSMC knockdown cells revealed strong co-localization. Panc-1 control cells showed a perinuclear staining of Nucleolin and VVL. Altogether the staining of Panc-1 control cells was much weaker compared to Panc-1 COSMC knockdown cells (Fig. 3c).

Previously, it was shown that two of the newly identified Tn antigen carrier proteins, Nucleolin and GRP-78 also localize at the plasma membrane [45, 46]. Therefore, we separated membrane-bound and cytosolic proteins of Panc-1 COSMC knockdown cells and control cells. Western blot analysis revealed that Nucleolin and GRP78 are membrane localized in pancreatic cancer cells, confirming previous findings. Furthermore, Annexin A2 and α-Enolase were also detected in membrane protein fractions of Panc-1 COSMC knockdown cells and control cells. Successful cellular fractionation was confirmed using antibodies targeting Her2 and GAPDH (Fig. 3d).

### COSMC knockdown promotes migration and survival and inhibits proliferation in PDAC cells in vitro.

**Fig. 4:**
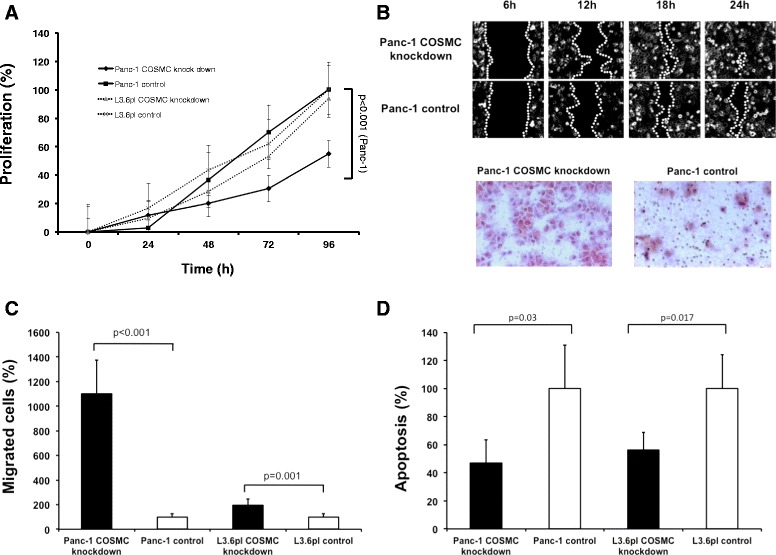
COSMC knockdown promotes migration and survival and inhibits proliferation in PDAC cells in vitro **a** Proliferation of Panc-1 COSMC knockdown cells compared to control. Proliferation was measured in MTT assay over 96 h using Panc-1 COSMC knockdown cells (*black*) and control cells (*grey*) (*p* < 0.001). L3.6pl COSMC knockdown and control cells are indicated as dotted line. **b** above: Scratch assay are displayed for Panc-1 COSMC knockdown cells compared to control at different time points. **b** below: HE stained transwell membranes after 24 h of Panc-1 COSMC knockdown cells (left) and control cells (right) **c**: Relative migration of Panc-1 and L3.6pl COSMC knockdown cells was compared to control cells using transwell assay. Migrated cells were determined after 24 h (*p* ≤ 0.001). **d**: Apoptosis rate of Panc-1 and L3.6pl COSMC knockdown cells was compared to control cells using ELISA based cleaved Caspase-3 assay. Cleaved Caspase-3 expression was determined after 24 h (*p* = 0.03 and 0 = 0.017)

To gain relevant insights into COSMC function in pancreatic cancer cells, we first analyzed whether COSMC might influence proliferation of Panc-1 cells. Interestingly, analysis of Panc-1 COSMC knockdown cells by MTT assay revealed a strong reduction in proliferation, compared to control cells (p <0.001), whereas COSMC knockdown had no effect on proliferation in L3.6pl cells (Fig. 4a). Next, we analyzed the influence of COSMC expression on cancer cell migration in vitro. Therefore, we performed transwell assays and quantified the migratory potential of COSMC knockdown as well as corresponding control cells after 24 h. Migration of Panc-1 COSMC knockdown cells was substantially higher compared to control cells (Fig. 4b-c) (*p* < 0.001). Additionally, migrating cells were recorded in a wound healing assay and the cell displacement rate during migration was quantified. The median displacement rate of Panc-1 COSMC knockdown cells was 154.4 μm/s whereas control cells showed a median displacement rate of 108.1 μm/s (*p* <0.05). The displacement rate of Panc-1 COSMC knockdown cells was 0.00153 μm/s and that of Panc-1 control cells was 0.00095 μm/s (*p* <0.05) (representative images are shown in Fig. 4b). The meandering index of Panc-1 COSMC knockdown cells was 0.261 and 0.196 in control cells (*p* <0.001). To analyze whether COSMC knockdown influences apoptosis in pancreatic cancer, a cleaved Caspase-3 ELISA assay was performed. Interestingly, COSMC depleted Panc-1 and L3.6pl cells showed substantially decreased apoptosis compared to corresponding control cells. Cleaved Caspase-3 expression was determined after 24 h and showed a 53 % reduced apoptosis rate in Panc-1 COSMC knockdown cells (*p* =0.03). L3.6pl COSMC knockdown cells showed a 44 % reduction of apoptosis rate (*p* =0.017) compared to control (Fig. 4d).

### Tn antigen and Nucleolin are frequently co-localized in PDAC patient specimens

It is well known that non-malignant epithelial cells display long branched O-linked glycan structures such as core 2 and core 3 glycans that are attached, for instance to various mucins [47]. In malignant cells, a complex interplay of various glycosyltransferases/chaperones promotes a reduced expression of long branched glycans and concomitant increase truncated glycans (Tn antigen).

**Fig. 5:**
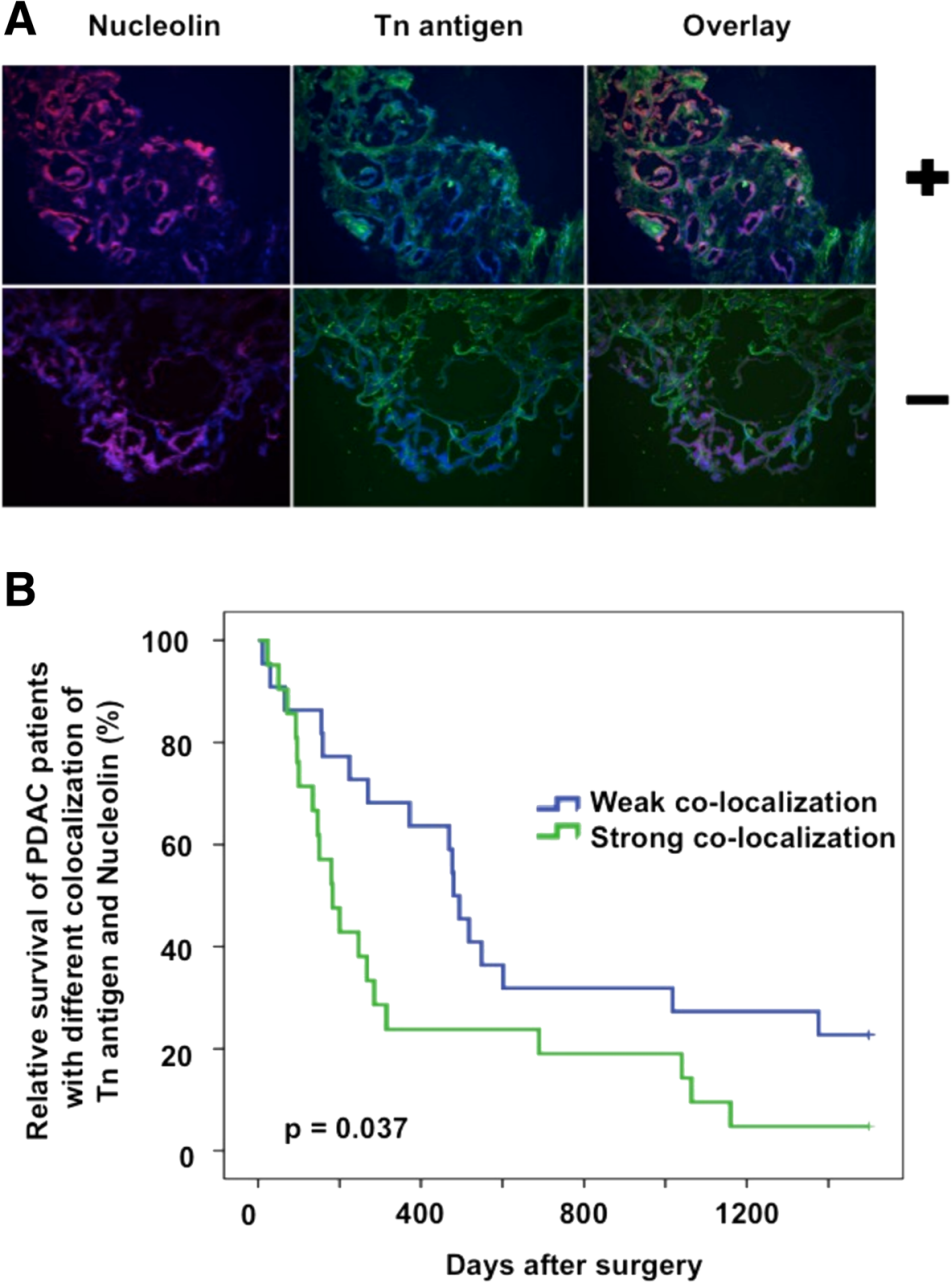
Tn antigen and Nucleolin are frequently co-localized in PDAC patient specimens. **a** Tn antigen and Nucleolin expression in PDAC. Immunohistochemistry of patient-derived PDAC samples revealed a frequent co-expression (orange) of Tn antigen using VVL-mediated staining (*green*) and Nucleolin staining (*red*). Nuclei were detected with DAPI (*blue*). Pictures marked with + displayed a tumor specimen with strong co-expression, whereas the tumor probe marked with – showed weak co-expression **b** Kaplan-Meyer survival plot with 43 PDAC patients with strong and weak co-localization of VVL and Nucleolin staining (*p* = 0.037)

To analyze whether pancreatic cancer cells display such truncated glycans on the MS-based identified Nucleolin, we analyzed 43 different PDAC patient specimens using VVL-mediated detection of Tn antigen and its possible co-localization with Nucleolin using specific antibodies. To exclude possible cross-reactivity of VVL with other blood group determinants (A and AB) [29], we solely analyzed PDAC specimens derived from non-A and non-AB blood group patients. The degree of Tn expression and Nucleolin expression partially reflects the degree of co-localization. In fact, co-localization was determined in 3 groups: 1. Tn/Nucleolin expression and co-localization, 2. Tn/Nucleolin expression without/minimal co-localization, 3. Minimal Tn/Nucleolin expression without/minimal co-localization. Group 1 was compared to group 2/3 and named strong co-localization vs. weak co-localization. Interestingly, immunofluorescence staining of these tumor specimens showed tumors with weak or strong co-localization of Tn antigen and Nucleolin (Fig. 5a). 22 (51 %) PDAC specimens showed weak and 21 (49 %) PDAC specimens showed strong co-localization. Moreover, correlation of this co-localization with available clinical survival data revealed poor survival for patients with a strong co-localization compared to patients with a weak co-localization of Nucleolin and VVL in Kaplan-Meyer survival analysis using Log-Rank test (*p* = 0.037). Five patients in the group of weak co-localization and one patient with strong co-localization of Tn antigen and Nucleolin were censored, because they outlived the 1500 days follow up periode (Fig. 5b). In our study, the degree of Tn antigen and Nucleolin expression is not related to survival of patients suffering pancreatic ductal adenocarcinoma (log-rank test, *p* = 0.95 and *p* = 0.34) (Additional file 1: Figure S10 and S11).

## Discussion

The discovery of Tn antigen was a milestone in the history of glycosciences [5]. The Tn antigen was first described in the Tn polyagglutination syndrome by Moreau *et al*. in a patient with hemolytic anemia [48]. The underlying mechanism was discovered 48 years later in 2005 by Ju and Cummings and was identified as a mutation of the COSMC chaperone [49]. Since then, functional studies with expression of Tn antigen via manipulation of COSMC chaperone was possible and the knowledge on differential cancer O-glycosylation was greatly expanded.

In this study, we examined different pancreatic cancer cell lines to give an overview of Tn antigen expression and to determine cell lines with low or moderate Tn antigen expression. We detected high molecular weight signals (150–250 kDa), indicating detection of high molecular weight proteins similar to the known mucin staining, but the Jurkat control cell line showed strong signals of lower molecular weight bands with approximately 75 kDa. On the other hand, lectins showed cell line dependent staining over a broad range with molecular weights between <50 - 250 kDa. These detected differences are probably due to varying binding specificity of different lectins and antibodies regarding the amino acid back bone of Tn antigen carrying proteins [50]. In addition, the detected Western blot signals are probably not solely Tn antigen expression but also a staining of terminal presented GalNAc structures [17–22, 51]. One way of Tn antigen expression in human carcinoma cells is the loss of functional COSMC. Tn antigen expression has been shown in cervical carcinoma, melanoma and colon carcinoma cells due to somatic mutation of COSMC or absence of COSMC transcript [15]. Epigentic silencing through methylation of COSMC promotor in pancreatic carcinoma caused inactivation of T-synthase with consecutive aberrant O-glycosylation [4]. In this study, we investigated the influence of Tn antigen expression on pancreatic carcinoma cells using a stable lentiviral-mediated COSMC knockdown and detected an enrichment of aberrant O-glycans using a broad array of antibodies and lectins.

Since GalNAc-transferases (GalNAc-Ts) catalyze the first step of O-glycosylation which results in the formation of Tn antigen, we also examined whether COSMC knockdown influences expression levels of all known human GalNAc-Ts. Interestingly, we detected substantially decreased mRNA levels for various GalNAc-Ts in COSMC knockdown cells. Down-regulation of GalNAc-T mRNA expression after COSMC knockdown might indicate a negative feedback regulation, which reduces the expression of GalNAc-T mRNA in the presence of elevated Tn antigen levels. This might represent a novel mechanism of regulation for the activity of GalNAc-Ts, since the activity of GalNAc-Ts is known to be regulated through expression levels or by regulated relocation of GalNAc-Ts from Golgi apparatus to endoplasmic reticulum [18]. Analyzing the obtained expression results of GalNAc-Ts, we can state with certainty, that the observed changes in O-glycosylation are COSMC knockdown mediated. In contrast, T-synthase mRNA expression levels increased 2-fold in COSMC knockdown cells compared to control cells. The increased T-synthase levels might be an attempt of the cell to overcome the lack of functional T-synthase.

Using mass spectrometry, we identified 23 different O-GalNAc glycosylated and abundantly expressed proteins in Panc-1 COSMC knockdown cells. Mapping of the human O-GalNAc glycoproteome has recently gained great importance using a zinc finger nuclease mediated targeting of COSMC gene [52]. Amongst several cell lines, this technology was also used in Capan-1, a pancreatic cancer cell line. The authors found that almost 50 % of the identified glycoproteins were expressed uniquely in one cell line and each cell line contributed its own repertoire of glycoproteins. Altogether only 14 proteins were identified in all 12 cell lines and a new subset of glycoproteins was found [52]. These findings suggest distinct O-glycoproteomes for each type of cancer, however, further investigations are needed to gain insights into its possible therapeutic value.

Targeting of tumor specific O-glycoslyation is a promising treatment strategy for cancer, but faces various challenges. After Tn antigen administration, a sufficient immune and anti-tumor response in nonhuman primates was induced [53]. Thereafter, Tn antigen evolved to an important component of glycoconjugate based anti-tumor vaccines [5, 54]. Springer and colleagues reported a successful vaccination of 16 breast cancer patients with a statistical significant better survival, however, the validity of their data cannot be determined due to the absence of several controls as well as appliance of a questionable glycoprotein preparation [55]. The treatment of prostate cancer was another focus of Tn antigen based vaccination. In a phase I clinical trial, all patients developed an immune response against Tn antigen, but only 33 % achieved favorable clinical responses with decreased detection of prostate-specific antigen [56]. Long-term survival data have not been published so far, but these studies might provide a first prospect whether Tn antigen vaccination results in favorable effects in patients. Nonetheless, to provide a substantiate basis for successful vaccination with Tn antigen, a proper antigen expression is required.

Since the first discovery of Tn antigen, a variety of methods have been developed, mostly being dependent on either antibodies or lectins. A high number of different GalNAc-binding lectins or antibodies are available and were already successfully applied in various expression studies of human cancers, including pancreatic cancer. Unfortunately, the results of these studies showed no consistency, which might be a consequence of different binding specificities of individually used lectins and antibodies. Tn antigen binding lectins and antibodies may also cross-react with other carbohydrate antigens [51]. In addition, monoclonal antibodies recognize not only the Tn antigen but also depend on the individual amino acid backbone of the protein, leading to a distinct recognition pattern of Tn containing glycoproteins on cancer cells [50].

Altogether, pancreatic cancer has most likely its specific glycoproteome and hence the need arises for its specific detection. Here, we focused on the identification of aberrant O-glycosylated proteins in pancreatic cancer. The proteins identified may contribute to a better understanding of the pancreatic cancer O-glycoproteome and may help to develop pancreatic cancer specific vaccinations or other therapies in future. Therefore, membrane-associated proteins were of pivotal interest, because they represent potential targets for immunotherapy. We evaluated all 23 O-glycoproteins regarding their localization in cancer, involvement in signal transduction and focused our analysis on four proteins for further characterization (Tab. 1). In this study, Tn antigen expression was confirmed for Nucleolin, GRP-78, Enolase and Annexin A2 using a VVL-based immunoprecipitation and all other identified Tn antigen carrier proteins were predicted to be highly O-glycosylated by the ISOGlyP database. Moreover, we could clearly show that Nucleolin is frequently co-localized with Tn antigen in patient-derived PDACs and strong co-localization is correlated with poor survival of the patients.

Additionally, the degree of Tn and Nucleolin expression is not related to survival of patients suffering from pancreatic ductal adenocarcinoma (log-rank test) (Additional file 1: Figure S8 and S9). To the best of our knowledge, the relation of Tn expression to survival was only reported for endometrial, colon and breast cancer [47, 57]. The lack of correlation in pancreatic cancer is difficult to explain and is possibly caused by the biologic nature of pancreatic cancer characterized by an extremely rapid tumor progression. Therefore, the survival of patients is generally very short and an influence of Tn antigen expression on survival is possibly masked.

Furthermore, information regarding the biological effects of COSMC knockdown on cell growth, migration and apoptosis in pancreatic cancer is scarce. Interestingly, our analysis of COSMC knockdown cells revealed an enhanced migration and decreased apoptosis accompanied by reduced proliferation compared to control cells in vitro. In breast cancer cells, implications of sialyl-Tn antigen on adhesion, migration, proliferation and tumor growth were already investigated and showed an enhanced tumorigenesis [58, 59]. Recently, Huang *et al*. detected an enhanced malignant behavior of colon cancer cells overexpressing COSMC chaperone [60], equivalent to our analysis of PDAC specimens, which showed an increased expression of COSMC and T-synthase (Additional file 1: Figure S10 and S11). Due to the large proportion of stromal tissue in PDAC, the obtained regulations in gene expression need not necessarily reflect the actual situation in cancer cells. Although in contrast to our findings, both overexpression and COSMC knockdown may lead to an enhanced expression of aberrant O-glycans and therefore contribute to the same malignant behavior of tumor cells.

We also analyzed Tn antigen and Nucleolin expression in pancreatic cancer specimens and found a poor survival of patients with strong co-localization of Nucleolin and VVL staining, which may suggest a new role of aberrant O-glycosylated proteins as biomarkers. Many of the current clinical biomarkers display carbohydrate modifications, but their basis and precise structure are largely not understood, because of their molecular complexity and their unknown regulations [61].

## Conclusion

In conclusion, our study demonstrates the influence of altered O-glycan (Tn/STn) expression on oncogenic properties in pancreatic cancer and identifies O-GalNAc modified Nucleolin as novel prognostic marker.

## Material and methods

### Cell culture

Panc-1, HEK-293 T, BxPC1 and MiaPaCa 2 cell lines were maintained in DMEM and L3.6pl were maintained in RPMI, each supplemented with 10 % (v/v) FCS, and 1 % (v/v) penicillin and streptomycin. Patient derived cell lines 5061, 5072 and 5167 were maintained in RPMI 1640 with Glutamax (Life Technologies) supplemented with 10 % of fetal calf serum (FCS), 200 IU/ml of penicillin-streptomycin, 0.1 mg/ml gentamycin (Biochrom), 50 nmol/ml of human transferrin (Sigma-Aldrich), 0.01 μg/ml of bovine insulin (Sigma-Aldrich), 0.01 μg/ml of recombinant human epidermal growth factor (Pepro Tech), and 0.01 μg/ml of human basic fibroblast growth factor (Pepro Tech).

Cells were cultured at 37 °C in a humidified atmosphere containing 5 % CO_2_. All cell lines were used at low passage number not exceeding 30 passages.

### Western blot analysis and antibodies/lectins

Total protein concentrations were measured using BCA Protein Assay (Thermo Fischer). Samples containing 30 μg total amount of protein were boiled for 5 min in Laemmli buffer and separated by SDS PAGE under reducing conditions in 4-15 % Mini-PROTEAN TGX gels (BIO-RAD). Proteins were blotted on nitrocellulose membrane (Thermo Fischer). The membrane was blocked with 1x Carbo-Free Blocking Solution (Biozol) in TBS-T and subsequently incubated with serum (1:20) in TBS-T overnight. Tn antigen antibody (MA1-80055, clone BRIC111, Thermo Fischer), sTn antigen antibody (ABIN356328, clone STN218, antibodies online) and COSMC antibody (19254-1-AP, Proteintech) were diluted 1:250 in TBS-T. HSC70 (sc-7298), GAPDH (sc-32233,) Nucleolin (sc-8031), GRP78 (sc-1051), Alpha Enolase (sc-7455) and Annexin II (sc-9061) antibodies were purchased from Santa Cruz and diluted 1:1000. Her2 antibody (#2165, Cell Signaling) was diluted 1:100. *Vicia villosa* lectin B4 (B-1235) (VVL) and *Wisteria floribunda* lectin (B-1355) (WFL) were purchased from Vector Laboratories and diluted 1:125. Washing was performed 5 times with TBS-T. The membrane was incubated with a specific HRP-antibody (1:1250; Life Technologies) for 1 h at RT. After 5 times washing with TBS-T buffer, immunodetection was performed using enhanced chemiluminescence (GE Healthcare). Protein expression was quantified using an LAS-3000 Imager from Fuji (Raytest, Straubenhardt, Germany).

### Lentiviral-mediated knockdown of COSMC chaperone

pLKO.1-puro vector encoding COSMC and non-target (scrambled, SCR) shRNA were purchased from Sigma-Aldrich (Germany). Generation of pseudotyped lentiviruses and transduction were performed as previously described [62]. Cells transduced with COSMC shRNA were selected by addition of puromycin (Sigma-Aldrich) to culture medium with a final concentration of 2 mM for at least one week.

### Immunoprecipitation and membrane protein extraction

Total cell lysates were prepared with RIPA buffer. Lysates (0,5 mg) were pre-cleared with 40 μl of protein A/G-agarose beads (Santa Cruz) for 1 h. Protein A/G-agarose beads were added to the afore mentioned antibodies and rotated at 4 °C 1 h. Pre-cleared proteins and bead-conjugated antibodies were mixed, rotated at 4 °C overnight, pelleted and washed five times with RIPA buffer. An equal volume of 6xLaemmli buffer was added, and samples were boiled and separated by SDS–polyacrylamide gel electrophoresis. For membrane protein extraction, harvested cells were resuspended in PBS and homogenized using a dounce homogenizer. Centrifugation at 100 g, 5 min, 4 °C removed undisrupted cells a second centrifugation at 100 g, 10 min, 4 °C extracted nuclei. After centrifugation at 20000 g for 90 min, at 4 °C, membranes were pelleted and the supernatant contained the post membrane fraction.

### Real-time PCR

Total RNA was isolated using the RNeasy Mini kit (Qiagen), reverse transcribed with Transcriptor reverse transcriptase (Roche) using an oligo-(dT) primer according to the manufacturer’s protocol. The cDNA was subjected to real-time PCR amplification using primer sets for COSMC, GalNAc-transferases, T-synthase and GAPDH (Additional file 2: Table S1). Real-time PCR was prepared with Maxima SYBR Green/ROX qPCR Master Mix, 0.3 μM forward and reverse primer and 750 ng cDNA and used on the Lightcycler 480 (Roche). The conditions were as follows: 95 °C for 10 min, 45 cycles at 95 °C for 15 s, and 60 °C for 1 min. CT values were determined using Lightcycler 480 Software version 1.5 (Roche). All samples were analyzed in duplicates, and values of the sample copies were obtained after quantitative amplification and normalized to GAPDH using 2^−^ΔΔ^CT^ method. Each experiment was at least repeated twice.

### Mass spectrometry (MS)

O-GalNAc modified proteins were identified by MALDI-MS. Protein bands were excised from Coomassie stained polyacrylamide gel and trypsin (sequencing grade, Promega) was added to each sample for 4 h at 37 °C. Extracted peptides were mixed with the matrix solution containing α-cyano-4-hydroxycinnamic acid, spotted for MALDI-MS measurement onto target slides, and analyzed using a matrix-assisted laser desorption/ionization time of flight (MALDI-TOF) mass spectrometer (Amersham Biosciences). All measurements were performed in the positive-ion reflective mode at an accelerating voltage of 23 kV and delayed-pulsed ion extraction. All peptide samples were measured as monoisotopic masses. Database searches were performed using the MASCOT software from Matrix Science with carboxymethylation of cysteine and methionine oxidations as variable modifications (probability value *p* <0.05).

### T-Synthase assay

Ten μg total protein from fresh cell lysates were directly used to determine endogenous T-synthase activity using UDP-Gal (Sigma Aldrich) as the donor and GalNAc-α-(4-MU) (Carbosynth) as the acceptor [63]. O-Glycosidase (New England Biolabs) was used for T antigen cleavage and release of fluorescent 4-MU. The 50 μL reaction system containing 1000 μM GalNAc-α-4-(MU), 500 μM UDP-Gal, 20 mM MnCl2, 0.2 % Triton X-100, 800 units of O-glycosidase, in 50 mM MES-NaOH buffer (pH 6.8), and 10 μg total protein was placed in a black OptiPlate-96 F (Perkin Elmer). Adding 100 μl 1 M Glycine NaOH (pH10) stopped reactions. Relative 4-MU fluorescence was measured (ex 355 nm/em 460 nm) in a FLUOstar Omega (BMG Labtech). The average value of the fluorescence intensities was calculated and the control cell line was set to 100 %. Error bars represent SEM.

### Proliferation, migration and apoptosis

The MTT assay was used for determination of cell proliferation. Cells were washed with PBS and MTT (Sigma) working solution was added into the wells. Cells were incubated at 37 **°** C for 2 h. Absorbance of the converted MTT dye was measured at a wavelength of 490 nm with FLUOstar Omega (BMG Labtech).

Cell migration was examined using transwell assay and scratch/wound healing assay. Briefly, 5 × 10^5^/mL cells were seeded onto a transwell insert (8-μm pore; BD Biosciences) using DMEM containing 0,1 % FBS. The fitted culture dishes were filled with DMEM containing 10 % FBS. Invaded cells were either stained after 24 h followed by four random views and quantification under a microscope or trypsinized and then quantified. For scratch assay, the corresponding cells were cultured to create a confluent monolayer. This monolayer was then scraped with a 200 μl pipet tip to create a “scratch” and wells were rinsed. The plate was then transferred a humidified incubation chamber, and cell migration was recorded using an Improvision LiveCell Spinning Disk system. Migrating cells were then tracked using Velocity 6.1.1. to quantify the respective cell displacement rate. Cell death was measured using Cleaved Caspase-3 sandwich ELISA (Cell Signaling) according to the manufacturer. Each experiment was repeated at least three times in quadruplicates.

### Immunofluorescence

Cells were cultured on coverslips, fixed with 4 % formaldehyde for 10 min, washed with PBS and afterwards blocked for 1 h in 1 % BSA/PBS-T at RT. The cells were then incubated with the primary antibody (Nucleolin 4E2, Abcam) over night (4 °C) or lectin (VVL-FITC, Vector) for 1 h at RT and washed with PBS. After incubation with the primary antibody, a fluorescent secondary antibody (Alexa Fluor 546 rabbit anti mouse IgG, Life Technologies) was added for 1 h at RT. After washing with PBS, the specimens were observed under a fluorescence microscope (BIOREVO BZ-9000; Keyence) and analyzed with BZ-9000 Generation II Analyzer software.

### Statistical analysis

Student’s *t*-Test (unpaired, 2-tailed) was calculated based on the data of at least three independent experiments. Bonferroni correction for multiple testing was performed where applicable. Results were considered significant if *p* <0.05. All error bars represent SD, unless indicated otherwise. Statistical survival analysis was performed using Kaplan-Meyer method. The differences between survival curves were tested using log rank test, *p* <0.05 was considered significant.

## Additional files

Additional file 1: Figure S1-S11. Differential expression of Tn antigen in human pancreatic carcinoma cell lines. Eight PDAC cell lines were available for analysis. Western blot was performed using HPA lectin (Fig. S1), VVL lectin (Fig. S2) and sialyl Tn antibody MA1-90577 (Fig. S3). HSC70 Western blot served as loading control. Jurkat cell line was used as positive control for Tn antigen expression. Figure S4: Lentiviral-mediated COSMC knockdown in Panc-1 cells. Western blotting total cell lysates of COSMC knockdown and corresponding control cells revealed successful down regulation of COSMC. HSC70 was used as loading control. Figure S5: Proteins of L3.6pl COSMC knockdown and control cells were used for Western blot with (right) and without (left) Neuraminidase treatment. Neuraminidase treated knockdown cells showed an enhanced Tn antigen signal, detected with VVL lectin, compared to controls. Figure S6: Immunocytochemistry was performed using VVL (green) and HPA (red) lectin for COSMC knockdown cells and control cells. Cell nuclei were counterstained using DAPI (blue). Figure S7: Distinct enrichment of aberrant O-glycans in Panc-1 COSMC knockdown cells. Protein precipitation with VVL lectin was performed. Precipitated proteins containing glyco epitopes were separated using SDS-PAGE and stained with Coomassie brilliant blue. Arrows indicate the further analyzed protein bands. Molecular weight is indicated in kDa. Figure S8 and S9: Kaplan-Meier survival estimation was calculated for patients with weak and strong Tn antigen expression (Fig. S8) and weak and strong Nucleolin expression (Fig. S9). No statistical differences were detected in both analyses (p = 0.95 and p = 0.34) using log-rank test. Figure S10: mRNA expression of COSMC and T-synthase in tumor (black bar) and control samples (white bar). Average Ct value for COSMC mRNA expression was 2.8 (±3.45) in PDACs and 5.24 (±1.27) in controls (p = 0.057). T-synthase expression levels showed an average Ct value of 4.8 (±2.45) in PDACs and 7.26 (±1.52) in controls (*p* = 0.015). ddCt values. Figure S11: Fold change of COSMC and T-synthase mRNA expression of tumor samples compared to controls are displayed, using the ΔΔCt method. Majority of the samples showed increased expression levels of analyzed genes in tumors compared to control. Additional file 2: Table S1. Real time PCR primer. Primer were designed using either Primer3 web interface [64] or taken from the Harvard PrimerBank [65].

## Abbreviations

PDAC: Pancreatic ductal adenocarcinoma
COSMC: *Co*re 1 β3-Gal-T-*s*pecific *m*olecular *c*haperone or C1GALT1C1
T-synthase: Core 1 β3-galactosyltransferase or C1GALT1
GalNAc: N-Acetylgalactosamine
MTT: 3-(4,5-dimethylthiazol-2-yl)-2,5-ditetrazolium bromide
VVL: *Vicia villosa* lectin
WFL: *Wisteria floribunda* agglutinin
