# Supplementary figures and images for: COSMC knockdown mediated aberrant O-glycosylation promotes oncogenic properties in pancreatic cancer
Source: Mol Cancer. 2015 May 29;14:109. doi: 10.1186/s12943-015-0386-1 (PMC4447007; doi:10.1186/s12943-015-0386-1)

Fig. S1

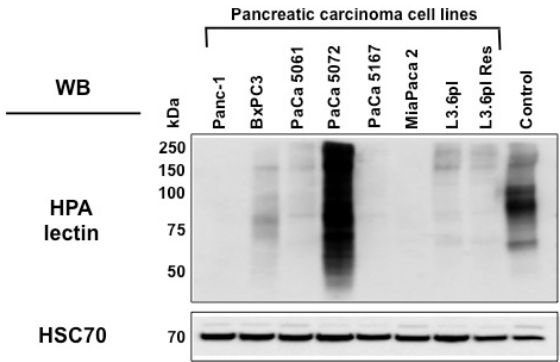

Fig. S2

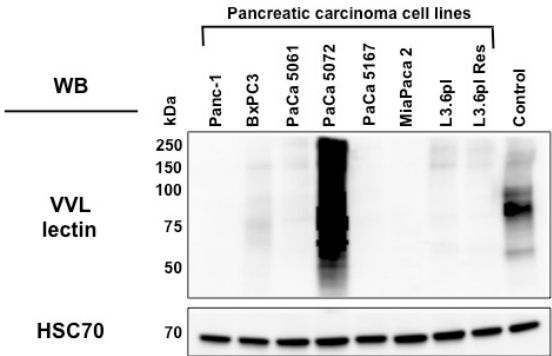

Fig. S3

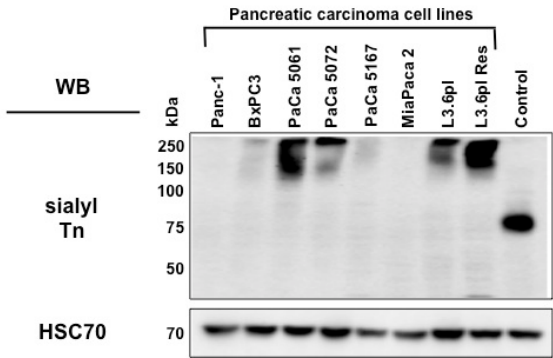

Fig. S4

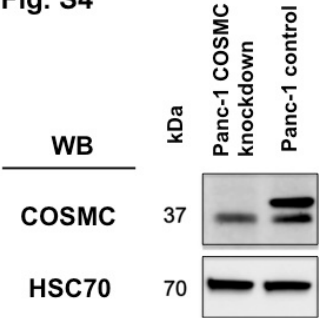

Fig. S5

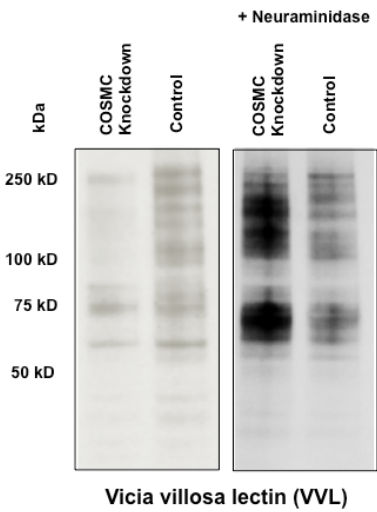

Fig. S6

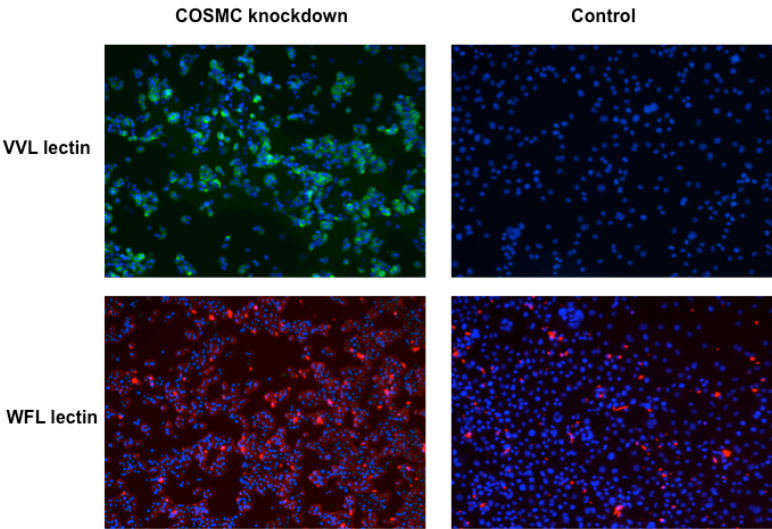

Fig. S7

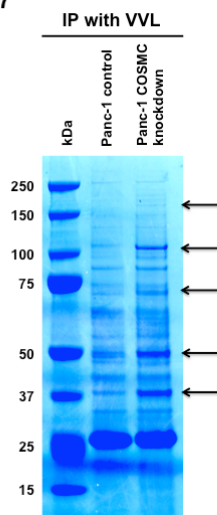

Fig. S8

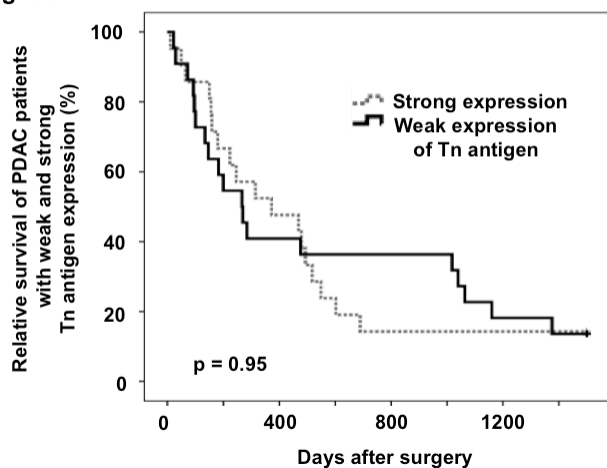

Fig. S9

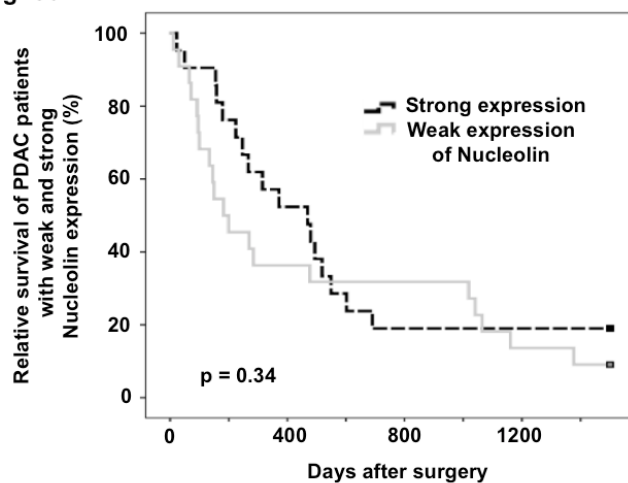

Fig. S10

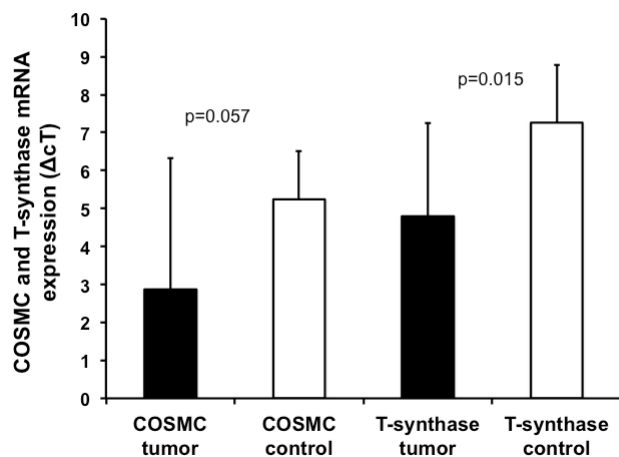

Fig. S11

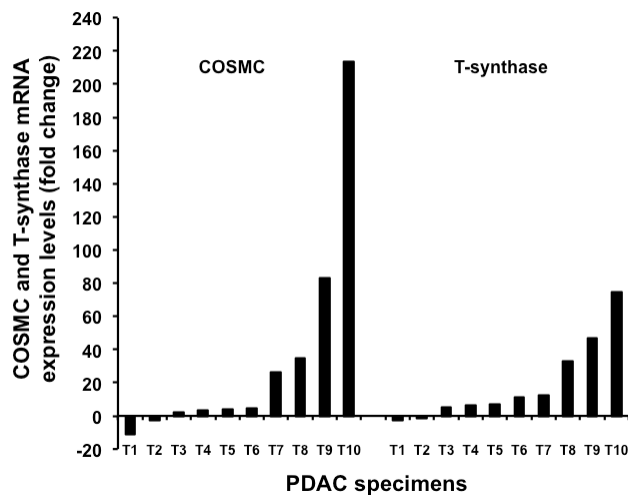

Supplement: Additional file 1: Figure S1-S11. — Differential expression of Tn antigen in human pancreatic carcinoma cell lines. Eight PDAC cell lines were available for analysis. Western blot was performed using HPA lectin (Fig. S1), VVL lectin (Fig. S2) and sialyl Tn antibody MA1-90577 (Fig. S3). HSC70 Western blot served as loading control. Jurkat cell line was used as positive control for Tn antigen expression. Figure S4: Lentiviral-mediated COSMC knockdown in Panc-1 cells. Western blotting total cell lysates of COSMC knockdown and corresponding control cells revealed successful down regulation of COSMC. HSC70 was used as loading control. Figure S5: Proteins of L3.6pl COSMC knockdown and control cells were used for Western blot with (right) and without (left) Neuraminidase treatment. Neuraminidase treated knockdown cells showed an enhanced Tn antigen signal, detected with VVL lectin, compared to controls. Figure S6: Immunocytochemistry was performed using VVL (green) and HPA (red) lectin for COSMC knockdown cells and control cells. Cell nuclei were counterstained using DAPI (blue). Figure S7: Distinct enrichment of aberrant O-glycans in Panc-1 COSMC knockdown cells. Protein precipitation with VVL lectin was performed. Precipitated proteins containing glyco epitopes were separated using SDS-PAGE and stained with Coomassie brilliant blue. Arrows indicate the further analyzed protein bands. Molecular weight is indicated in kDa. Figure S8 and S9: Kaplan-Meier survival estimation was calculated for patients with weak and strong Tn antigen expression (Fig. S8) and weak and strong Nucleolin expression (Fig. S9). No statistical differences were detected in both analyses (p = 0.95 and p = 0.34) using log-rank test. Figure S10: mRNA expression of COSMC and T-synthase in tumor (black bar) and control samples (white bar). Average Ct value for COSMC mRNA expression was 2.8 (±3.45) in PDACs and 5.24 (±1.27) in controls (p = 0.057). T-synthase expression levels showed an average Ct value of 4.8 (±2.45) in PDACs and 7.26 (±1. [file 12943_2015_386_MOESM1_ESM.pdf]

Fig. S7

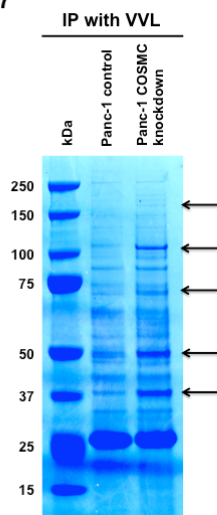

Fig. S8

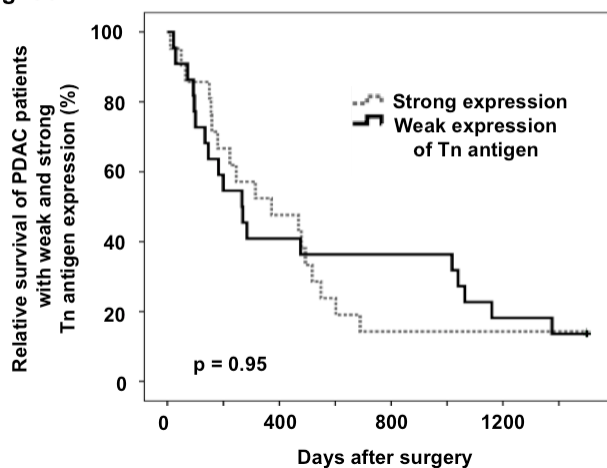

Fig. S9

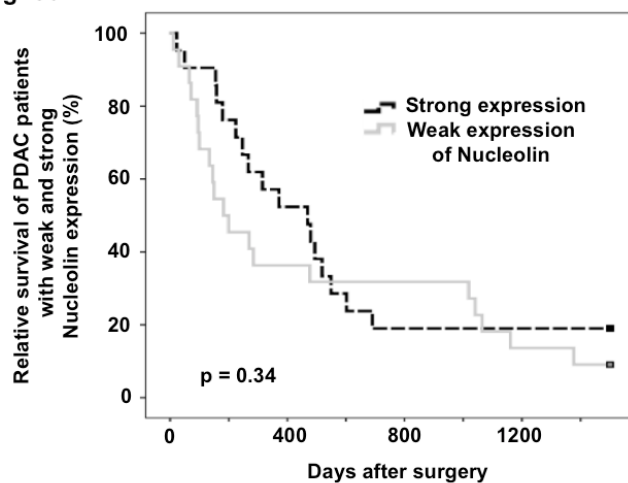

Fig. S10

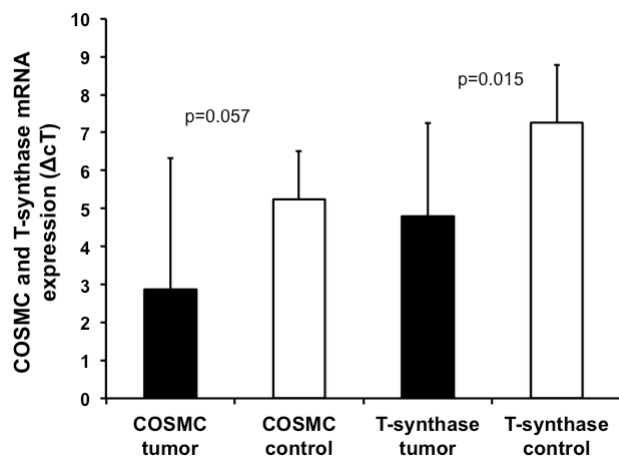

Fig. S11

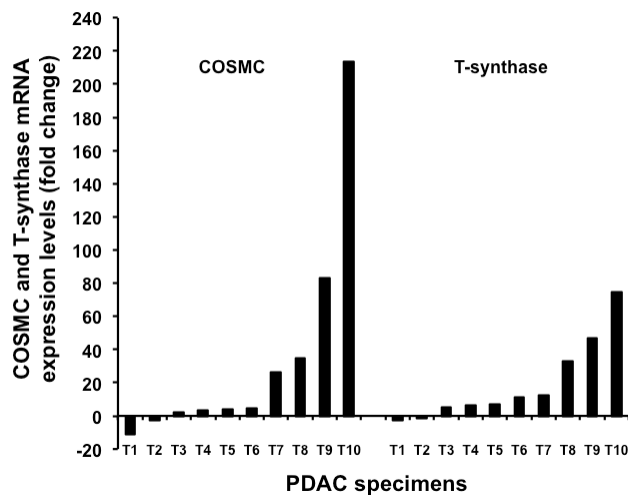

Supplement: Additional file 2: Table S1. — Real time PCR primer. Primer were designed using either Primer3 web interface [64] or taken from the Harvard PrimerBank [65]. [file 12943_2015_386_MOESM2_ESM.docx]
